# From eScience to iScience “I want Answers not Links” – new ways to search the Internet

**DOI:** 10.1186/1758-2946-4-S1-P22

**Published:** 2012-05-01

**Authors:** AJ Kos, D Steinen

**Affiliations:** 1 Dr. Alexander Kos, AKos GmbH, Austr. 26, D-79585 Steinen, Germany

## 

The industry uses the word eScience to illustrate that in most parts of science everything is done with computers. In chemistry, computer simulations and all the multitude of software applications has reduced the need for many experiments. This is good in a world of diminishing resources. But one application is slow to get a foothold in academia, it is the electronic lab journal. This is very unfortunate because this application is probably the one that makes writing publications and Ph.D. theses much quicker. This is a personal benefit, and a global benefit is that it makes accessible all these experiments that did not work, or did not make it into publication. This information is by far the largest amount of knowledge, and this is wasted, today. This an area where we need to catch up.

Another area where chemists need to rethink is how to access the wealth of information in the Internet. We chemist are all using Google or similar search engines, but we cannot search in the language of the chemists, the chemical structure. CWM Global Search solves this problem. It allows searching Google and more than 50 other major Internet sources by structure, CAS Registry Number, name and identifier. This is very good, but what we get are still links. From there we have to click many times until we finally find an answer. Do you sometimes limit your search to Wikipedia, because you can quickly and directly find an answer? But, Wikipedia is not the Internet. For scientist the answer is often not a single fact, but a table, or even better a graph that visualizes the data. It seems this vision is far in the future.

We cannot solve everything, but with Pipeline Pilot and similar workflow programs, we can build solutions that extract data from the link pages.

The Documents and Text Collection (DTC) for Pipeline Pilot focuses on finding, analyzing, and displaying information from text and documents. A key use of the DTC is to enhance and support the analysis of experimental data generated during your research projects. To do this, the DTC offers broad capabilities, including the ability to search and retrieve documents from online (e.g., PubMed, US & WO Patents) and local (e.g., SharePoint) repositories, and to crawl web sites to gather supporting data and competitive intelligence. For deeper text analytic methods DTC also integrate with 3rd party text analytics applications and technologies such as Linguamatics I2E and the UIMA framework.

